# Knockdown of miR‐19a suppresses gastrointestinal dysmotility diarrhea after TBI by regulating VIP expression

**DOI:** 10.1002/brb3.3071

**Published:** 2023-05-22

**Authors:** Ying An, Sheng Hu, Yu Zhang, Zhengji Song, Ruochang Li, Yan Li, Yongli Li, Wenjun Ren, Ping Wan

**Affiliations:** ^1^ Department of Gastroenterology The First People's Hospital of Yunnan Province/The Affiliated Hospital of Kunming University of Science and Technology Kunming Yunnan China; ^2^ Department of Hepatobiliary Surgery The Second Affiliated Hospital of Kunming Medical University Kunming China; ^3^ Department of Thoracic Surgery The First People's Hospital of Yunnan Province/The Affiliated Hospital of Kunming University of Science and Technology Kunming Yunnan China

**Keywords:** diarrhea, miR‐19a, traumatic brain injury, VIP–NO–cGMP–PKG pathway

## Abstract

**Background and aims:**

Traumatic brain injury (TBI) is the main cause of death and can lead to a variety of physiological complications, including gastrointestinal dysfunction. The present study aimed to confirm the miR‐19a‐mediated suppression of diarrhea after TBI through the regulation of VIP expression.

**Methods:**

A rat model of TBI induced by controlled cortical injury was used to observe gastrointestinal morphology by opening the abdomen after TBI. After 72 h of injury, the fecal water content of the rats was measured. The end ileal segments were removed, and HE staining was used to observe the histopathological changes in the intestine. The levels of serum miR‐19a and VIP mRNA were detected by qRT‐PCR. ELISA was performed to detect VIP levels in serum. Immunohistochemistry was used to detect the level of VIP in ileal tissues, and immunofluorescence was used to detect c‐kit expression in ileal tissue. CCK‐8 assay was used to detect the cell viability of interstitial cells of Cajal (ICCs), and TUNEL assay was used to detect apoptosis of ICCs.

**Results:**

miR‐19a and VIP were highly expressed in the serum of TBI rats, and the knockdown of miR‐19a alleviated TBI‐induced diarrhea. In addition, the overexpression of miR‐19a or VIP inhibited the proliferation of ICCs, promoted apoptosis, and suppressed intracellular Ca^2+^ levels, whereas miR‐19a suppression had the opposite effects. A nonselective nitric oxide synthase inhibitor (L‐NA), PKG inhibitors (KT‐5823 and RP‐8CPT‐cGMPS), and a guanylate cyclase inhibitor (ODQ) restored the inhibitory effects of VIP on ICC proliferation, anti‐apoptosis effects, and Ca^2+^ concentrations.

**Conclusion:**

Knockdown of miR‐19a inhibits activation of the VIP–NO–cGMP–PKG pathway through suppression of VIP expression, which in turn inhibits diarrhea after TBI.

## Graphical abstract

Traumatic brain injury (TBI) is the main cause of death and can lead to diarrhea. Knockdown of miR‐19a inhibits activation of the VIP‐NO‐cGMP‐PKG pathway through suppression of VIP expression, which in turn inhibits diarrhea after TBI.

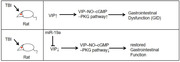

## 1 INTRODUCTION

In adolescents and children, traumatic brain injury (TBI) is the main cause of death and is a major health problem worldwide (Hu et al., 2022; Khalin et al., 2022), accounting for approximately 70% of traumatic deaths (Li et al., 2022). There is increasing evidence that TBI leads to a variety of physiological complications, among which gastrointestinal dysfunction (GID) is the major complication of TBI with a prevalence of up to 80% (Fu et al., 2020). According to the literature, GID after TBI mainly includes intestinal mucosal disruption, barrier dysfunction, and intestinal bacterial and endotoxin translocation as well as upper gastrointestinal bleeding (Cheng et al., 2018). GID is characterized by delayed intestinal contractile activity (Sun et al., 2015), gastroesophageal reflux (Olsen et al., 2013), gastroesophageal reflux, and intolerance to food (vomiting, bloating, and diarrhea) (Zhang et al., 2017). GID leads to nutritional deficiencies and abnormal drug absorption in patients with TBI, which affects the overall prognosis (Olsen et al., 2013; Sun et al., 2015; Zhang et al., 2017). Diarrhea is considered to be the most common symptom of food intolerance in patients with TBI (Vieira et al., 2018). Thus, it is important to understand how to improve diarrhea after TBI.

As a neuropeptide consisting of 28 amino acids, vasoactive intestinal peptide is closely related to the occurrence and development of many clinical diseases, especially gastrointestinal motility disorders (Fahrenkrug, 2010; Tomita, 2009). It has been reported that VIP is secreted by intestinal neurons and signals through vasoactive intestinal peptide receptor type 1 (VPAC1) on epithelial cells to stimulate the production of cyclic adenosine monophosphate in intestinal epithelial cells, thus inducing the secretion of water and electrolytes (especially potassium and bicarbonate), which enter the intestinal lumen with water and regulate ion and water homeostasis in the intestine (Mccauley et al., 2020; Thivacaren et al., 2022). Hang et al. (2004) reported that VIP levels in blood and ileal tissue significantly increase and reach the highest level 72 h after TBI in rats, suggesting that VIP may play an important role in diarrhea after TBI.

Several studies have found that some miRNAs regulate the expression of VIP or VIP receptors. Upregulation and downregulation of numerous miRNAs affect the release of VIP neurotransmitters in peripheral nerve injury (Musumeci et al., 2018). For example, miR‐525‐5p regulates the level of VPAC1 by targeting the 3′UTR of VPAC1 (Cong et al., 2021). In addition, miRNA‐19a expression is elevated in the serum of rats with functional dyspepsia, and VIP levels are simultaneously increased. Reducing miRNA‐19a expression results in lower VIP levels and improved gastrointestinal motility in rats (Deng et al., 2018). Thus, VIP levels are correlated with miRNA‐19a expression, and miR‐19a regulates VIP expression levels to affect gastrointestinal motility. However, it remains unknown whether miR‐19a plays a role in diarrhea after TBI.

Interstitial cells of Cajal (ICCs) are a special class of interstitial cells in the gastrointestinal tract. ICCs are the pacemakers and transmitters of gastrointestinal slow‐wave potentials, and they are mainly involved in the regulation of basic gastrointestinal electrical rhythms and neurotransmitter signaling, regulating gastrointestinal motility patterns (Daniel, 2010; Foong et al., 2020; Singh et al., 2021; Yadak et al., 2019). The main cause of GID is the change in the structure, function, number, and distribution of ICCs (Liu et al., 2021). Therefore, it is important to study the causes of the abnormal structure, function, quantity, and distribution of ICCs to understand GID diarrhea after TBI. Studies have shown that the nitric oxide–cyclic guanosine monophosphate (NO–cGMP) pathway is associated with gastric emptying in rats (Akm et al., 2018) and that it plays an inhibitory role in the pacing potential of mouse small intestinal ICCs (Kim et al., 2018). In contrast, VIP inhibits the pacemaker activity of ICCs via the NO–cGMP–PKG pathway (Kim et al., 2006).

Therefore, the present study aimed to investigate whether miR‐19a affects the number of ICCs and pacing potential through the regulation of the VIP‐NO‐cGMP‐PKG pathway, thereby affecting diarrhea after TBI.

## 2 MATERIALS AND METHODS

### 2.1 TBI rat model establishment

In total, 40 male standard deviation (SD) rats (12–16 weeks, weighing 180–220 g) were randomly divided into the following groups: sham‐operated group (sham group, 10 rats), TBI model group (TBI group, 20 rats), and miR‐19a inhibitor‐treated group (TBI + miR‐19a inhibitor group, 10 rats). The TBI model was induced in rats using the controlled cortical injury (CCI) model according to a previous report (Li et al., 2020; Zheng et al., 2018). Briefly, 5% isoflurane was used to anesthetize rats through inhalation, and the rats were placed in a stereotactic frame. An incision was made in the skin of the skull vault, and a circular craniotomy (5 mm in diameter) was performed between the fontanelle and λ with a ring drill. With the help of a Traumatic Brain Injury Impactor TBI 0310 (J&K Seiko, China), rats were then exposed to unilateral moderate CCI for 500 ms with a depth of 2.0 mm and a retention time of 3.5 m/s. Rats in the sham group received the same surgical procedure except for the CCI injury. In the TBI + miR‐19a inhibitor group, the miR‐19a inhibitor was administered by intraperitoneal injection. After successful transfection, a TBI rat model was established by CCI.

### 2.2 Morris water maze (MWM) experiment

Ten TBI rats were randomly selected and subjected to Morris water maze (MWM) experiments on Day 7 after CCI according to a previous study (Li et al., 2020). Briefly, rats were placed at different locations in a cylindrical pool (85 cm diameter and 60 cm high) with water (30 cm depth) and allowed to reach a visible platform placed in the center of the target area within 60 s in a positioning navigation test. If the rats did not find the platform, they were guided and boarded the platform and stayed for 10 s. This experiment was performed four times a day for 5 days with an experimental interval of 4 min. On Day 6, the platform was removed from the pool, and the rats were placed in the quadrant opposite to the target quadrant and swam freely for 60 s. Trajectory images were captured using ANY‐maze software to record escape latency and the platform crossed times. The experiment was performed by an operator who was unaware of the grouping.

### 2.3 Fecal water content

After TBI injury, 10 pellets of fresh rat feces were collected at 3, 12, 24, 48, and 72 h, and the wet weight was recorded. The pellets were then dried in an oven at 100°C for 3 h to obtain the dry weight. The percentage of water in the feces was calculated as (𝐴 − 𝐵)/𝐴×100%.

### 2.4 Histological analysis

The terminal ileal segment was removed 72 h after injury, and 4% paraformaldehyde was used to fix the intestinal samples for 2 h. The samples were then embedded in paraffin and cut into 5‐μm sections. After routine dewaxing and dehydration, the sections were stained with hematoxylin and eosin to observe the morphological changes in the intestinal tissues. The tissue sections were then blocked with 5% goat serum for 1 h. The number of ICCs and grid number were detected by immunofluorescence according to previous reports (Sun et al., 2015), and VIP expression was examined by immunohistochemistry (Jiang et al., 2017).

### 2.5 Cell culture and transfection

ICCs were purchased and cultured in cardiomyocyte medium with fetal bovine serum (5%) and streptomycin (1%) in an incubator at 37°C and 5% CO_2_. The smooth muscle growth medium was supplemented with 5 ng/mL rat stem cell factor. Lipofectamine 2000 reagent (Invitrogen, Carlsbad, CA, USA) was used for cell transfection according to manufacturer's instructions. miR‐19a mimic, miR‐19a inhibitor and negative control, and si‐VIP and VIP overexpression plasmids were transfected into cells. Cells were collected 24 h after transfection for follow‐up experiments.

### 2.6 Cell treatment

Cells were inoculated in 6‐well plate and when the cells were filled to 60%–70%, nonselective nitric oxide synthase (NOS) inhibitor L‐NA (100 mM), PKG inhibitor KT‐5823 (1 mM) and RP‐8CPT‐cGMPS (10 mM), and guanylate cyclase inhibitor ODQ (100 mM) were co‐incubated with ICCs cells, respectively.

### 2.7 qRT‐PCR

Total RNA was extracted from ICCs using a Total RNA Extractor (Sangon Biotech), and a cDNA synthesis kit (Vazyme, Nanjing, China) was used to reverse transcribe 2 μg of mRNA into cDNA, which was then diluted 10‐fold. The qRT‐PCR assay used 1 μL of prepared cDNA, and β‐actin and U6 were used as references. All primers used in this study were designed with Premier 5.0. The confidence of the PCR results was assessed by a dissociation curve and cycle threshold values. The results were calculated by the 2^−ΔΔ^ *^Ct^* method, and the experiment was repeated at least three times.

### 2.8 ELISA

The culture supernatants were collected and used to detect VIP levels using ELISA kits (MLBIO, Shanghai, China). In brief, 100 μL of lysate was added to the ELISA plates for 2 h, and corresponding antibodies were then added to the plates followed by incubation for 1 h. After washing the ELISA plates, they were incubated for 20 min with horseradish peroxidase‐streptavidin, and the absorbance values were measured at 450 nm by a microplate spectrophotometer.

### 2.9 CCK‐8 assay

ICCs were seeded into 96‐well plates (100 μL/well) and cultured for 24 h in a 37°C incubator. After transfection or dosing, 10 μL of CCK‐8 reagent was added followed by incubation for 2 h. An enzyme marker (ELX800, BioTeK, UK) was then used to measure the absorbance at 450 nm. Each experiment was repeated three times independently.

### 2.10 TUNEL assay

According to the TUNEL kit (Beyotime, Shanghai, China), cells at the logarithmic growth stage were digested with trypsin, centrifuged, washed with PBS, and fixed with 4% paraformaldehyde at 25°C for 30 min. Subsequently, cells were blocked with a 0.3% H_2_O_2_ methanol solution, and cells were then incubated with the TUNEL assay solution. Cells were then washed with PBS and sealed with anti‐fluorescence quenching–blocking solution. Cells and tissues were imaged under a fluorescence microscope at a wavelength of 450–500 nm (400857, Nikon, Japan). PI was used to stain apoptotic cells red, and DAPI was used to stain nonapoptotic cells blue. Green fluorescence indicated FITC‐12‐dUTP in the nuclei of apoptotic cells.

### 2.11 Fluo‐3 AM assays for cellular Ca^2+^ concentration

ICCs were incubated with 5 μM Fluo‐3 AM at 37°C for 30 min in the dark. An LSM510 confocal laser scanning microscope (excitation wavelength of 488 nm and emission wavelength of 530 nm) was used to observe the fluorescence intensity. Binding of intracellular free Ca^2+^ to Fluo‐3 AM is indicated by green fluorescence.

### 2.12 Statistical analysis

GraphPad Prism 8 software was used to analyze and prepare graphs. Data are presented as the mean ± SD. Unpaired one‐way analysis was used to analyze multiple groups, and Student's *t*‐test was used to analyze two groups. *p* < .05 was considered statistically significant.

## 3 RESULTS

### 3.1 Successful establishment of the TBI rat model

**FIGURE 1:**
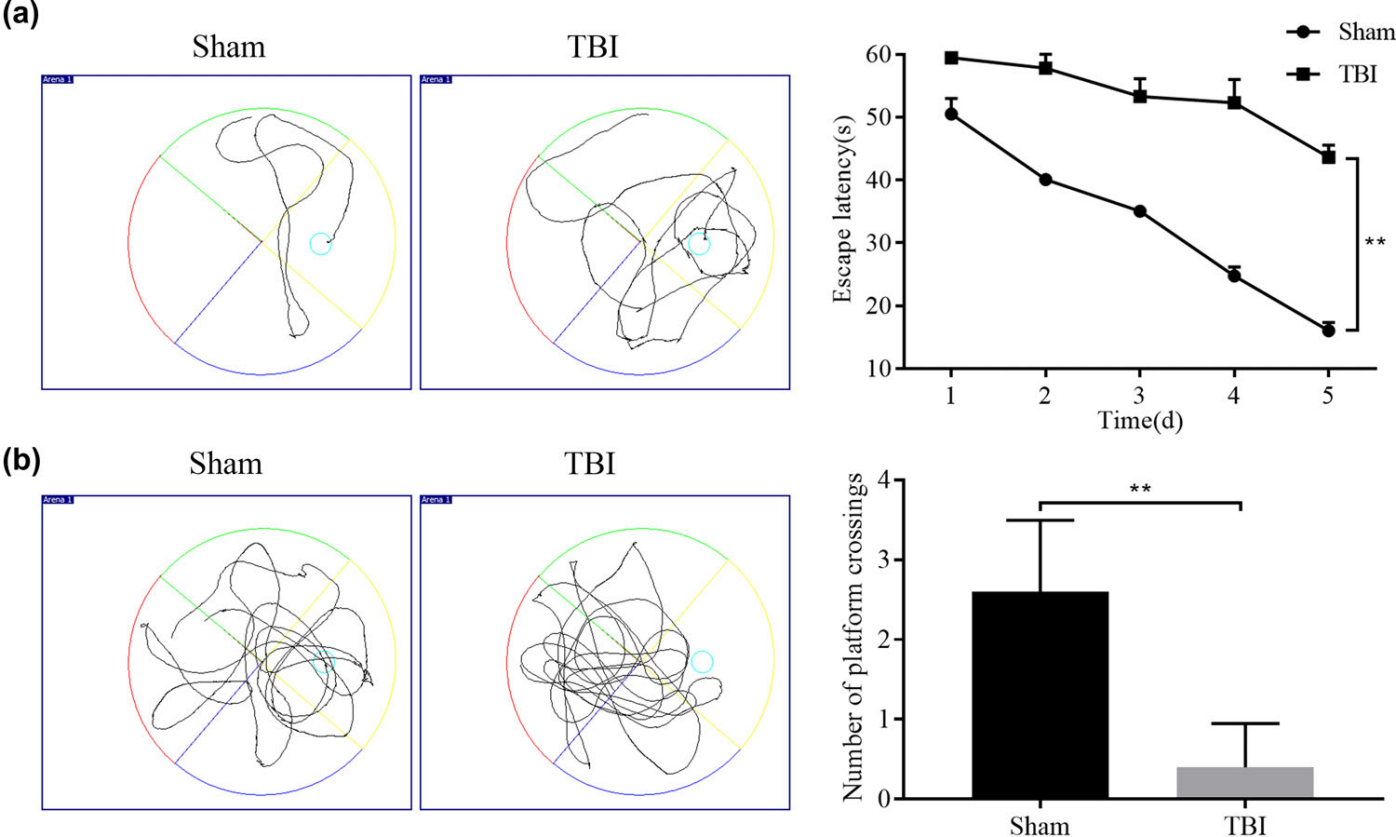
Morris water maze (MWM) experiment to test the effect of the model: (A) representative plots of trajectories and quantitative analysis results of escape latency in rats in the MWM experiment; (B) representative plots of trajectories and quantitative analysis results of the number of platform crossings in the MWM test. ^*^ *p <* .05 and ^**^ *p <* .01.

To assess the learning memory capacity of the injured rats to determine the modeling efficiency, the MWM experiment was performed. The TBI rats showed a longer escape latency (Figure 1A) and a significantly lower number of platform crossings (Figure 1B) compared to the sham group, demonstrating successful modeling.

### 3.2 Significant diarrhea and abnormal expression of miR‐19a and VIP in TBI rats

**FIGURE 2:**
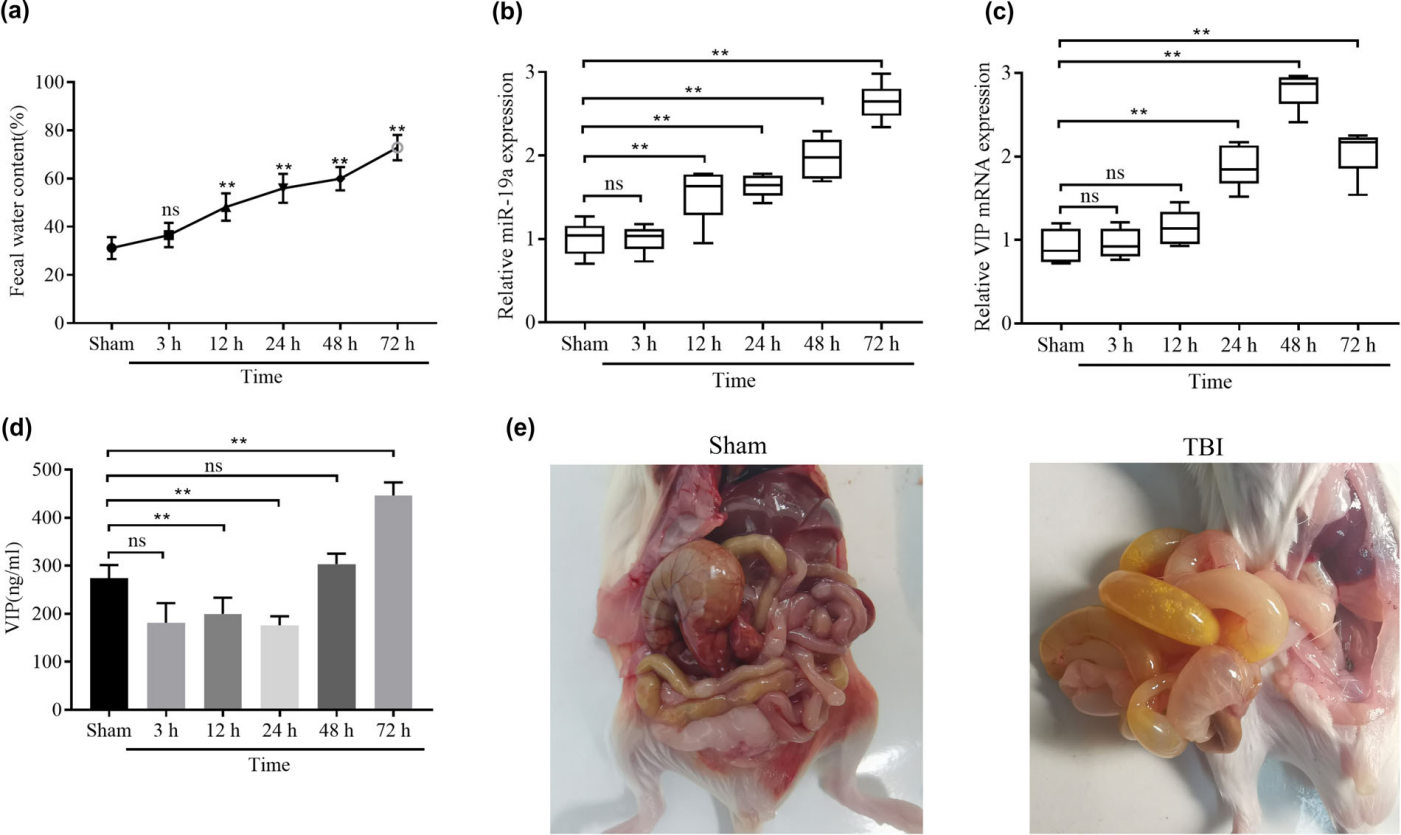
Traumatic brain injury (TBI) rats have diarrhea and abnormal expression of miR‐19a and VIP: (A) rat fecal water content assay, and RT‐qPCR analysis of serum miR‐19a (B) and VIP mRNA (C) expression levels; (D) ELISA analysis of serum VIP levels; (E) macroscopic map of rat gastrointestinal tract morphology. ^*^ *p <* .05 and ^**^ *p <* .01.

At 3, 12, 24, 48, and 72 h after TBI, the fecal water content of rats was evaluated, which demonstrated that the fecal water content of the TBI group was significantly increased after 12 h (Figure 2A). The level of serum miR‐19a was upregulated at 12 h after TBI and peaked at 72 h. At 24 h after TBI, the level of serum VIP mRNA was upregulated and peaked at 48 h (Figure 2B,C). ELISA was used to detect the serum VIP levels. After TBI, the rat serum VIP levels showed a decreasing trend at 3, 12, and 24 h, but the VIP levels slightly increased at 48 h. At 72 h after TBI, the rat serum VIP levels were significantly upregulated (Figure 2D). At 72 h after TBI, the abdominal cavity was opened to observe the morphology of the gastrointestinal tract, and it was found that the gastrointestinal tract of TBI rats was significantly dilated, accompanied by a large amount of yellowish fluid and gastrointestinal wall edema (Figure 2E). Thus, these findings indicated that rats with TBI had diarrhea and abnormal levels of miR‐19a and VIP.

### 3.3 Effect of miR‐19a overexpression/knockdown on VIP expression and ICC proliferation, apoptosis, and Ca^2+^ levels

**FIGURE 3:**
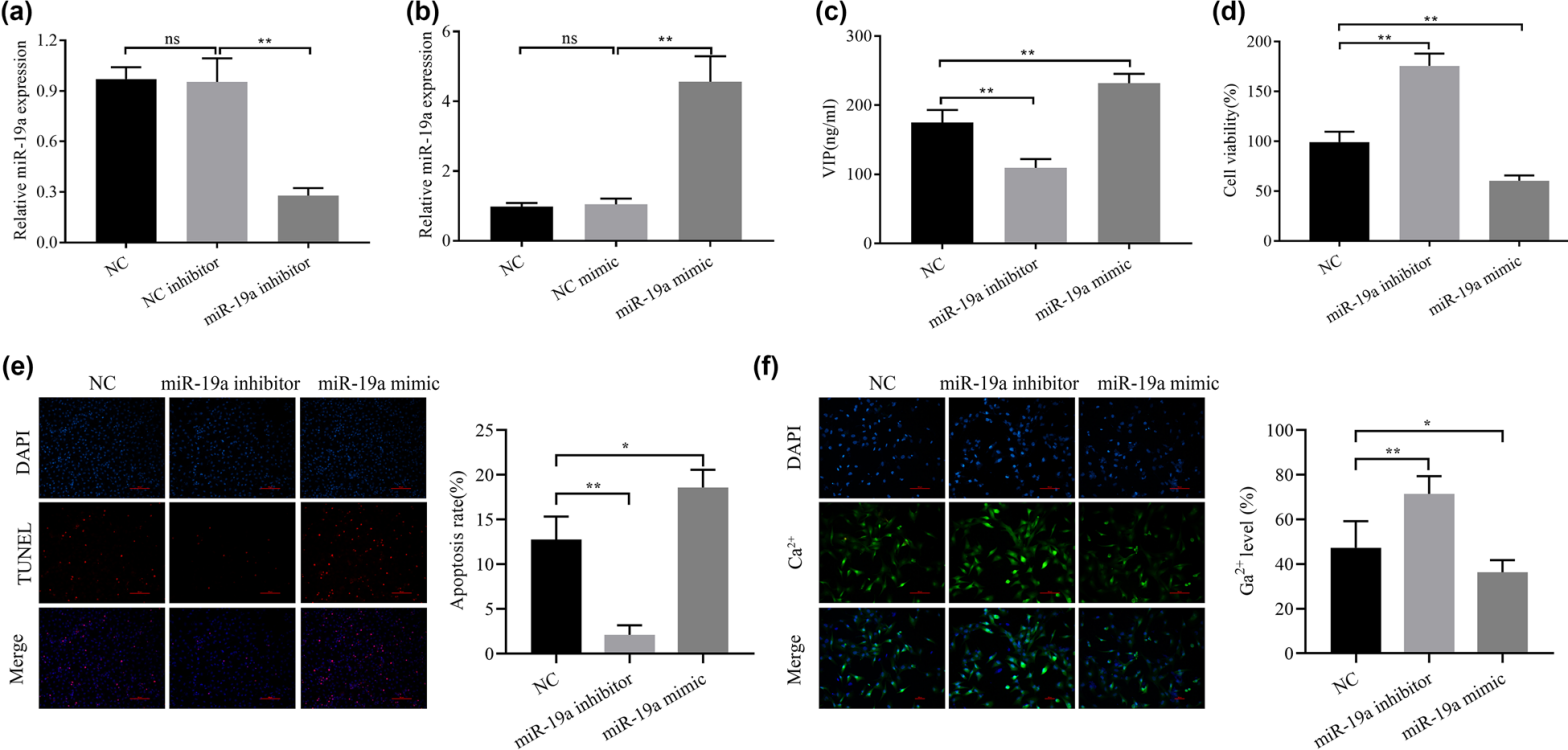
miR‐19a affects the proliferation, apoptosis, and Ca^2+^ levels of interstitial cells of Cajal (ICCs). qRT‐PCR analysis of miR‐19a expression after transfection with miR‐19a mimic (A) and miR‐19a inhibitor (B); (C) ELISA analysis of VIP levels; (D) CCK‐8 assay was used to detect cell viability; (E) TUNEL assay was used to detect apoptosis; (F) Fluo‐3 AM assay was used to detect cellular Ca^2+^ concentration. ^*^ *p <* .05 and ^**^ *p <* .01.

To detect the influence of miR‐19a expression on ICCs, an miR‐19a mimic and miR‐19a inhibitor were transfected into cells to alter the levels of miR‐19a expression. qRT‐PCR analysis demonstrated that the miR‐19a inhibitor significantly decreased the miR‐19a levels (Figure 3A), and that the miR‐19a mimic significantly increased the miR‐19a levels (Figure 3B). Moreover, the VIP levels were significantly decreased by miR‐19a downregulation and increased by miR‐19a upregulation as detected by ELISA (Figure 3C). The CCK‐8 assay indicated that the ICC viability was significantly increased by miR‐19a downregulation but that the ICC viability was decreased by miR‐19a upregulation (Figure 3D). The TUNEL assay showed that miR‐19a downregulation led to decreased ICC apoptosis and that miR‐19a upregulation led to increased ICC apoptosis (Figure 3E). Because the gastrointestinal motility pattern is initiated by the pacemaker activity of ICCs and intestinal motor neurons from neural input are induced (Singh et al., 2021), Ca^2+^ transients induce pacemaker depolarization in ICCs (Youm et al., 2019). Therefore, we investigated the influence of miR‐19a on Ca^2+^ levels in ICCs. The Ca^2+^ levels in ICCs were decreased by miR‐19a overexpression but increased by miR‐19a downregulation. In addition, the cellular Ca^2+^ concentration was detected using Fluo‐3 AM, which demonstrated that the Ca^2+^ concentration in ICCs was significantly increased by miR‐19a downregulation but significantly decreased by miR‐19a overexpression (Figure 3F). These results indicated that low miR‐19a levels promote ICC proliferation, anti‐apoptosis effects, and Ca^2+^ concentrations but that high miR‐19a levels have an inhibitory effect.

### 3.4 Overexpression of miR‐19a inhibits ICC activity through upregulation of VIP

**FIGURE 4:**
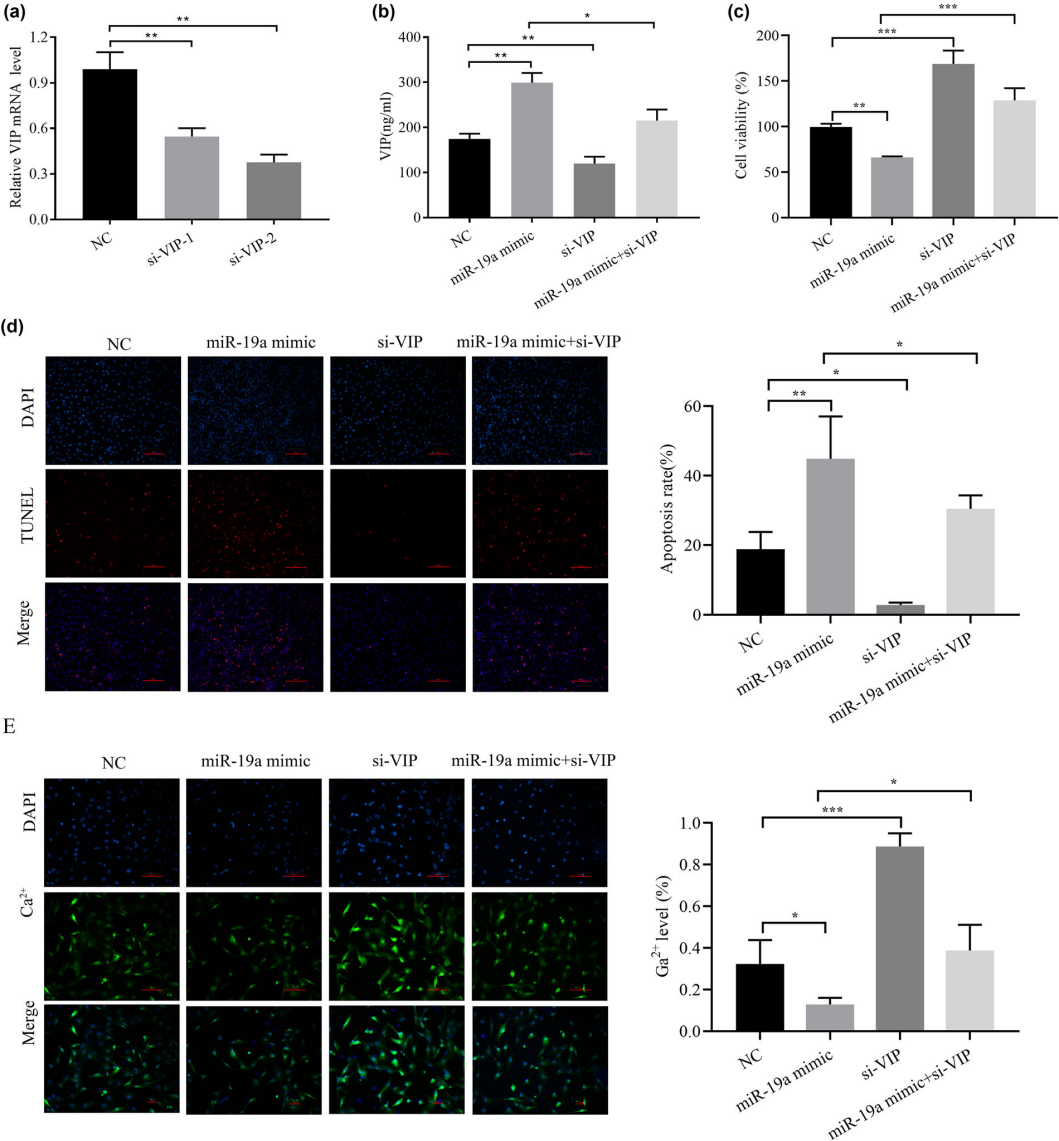
miR‐19a affects interstitial cells of Cajal (ICCs) by regulating VIP: (A) qRT‐PCR analysis of VIP levels after transfection of si‐VIP‐1 and si‐VIP‐2; (B) ELISA analysis of VIP levels; (C) detection of cell viability by CCK‐8 assay; (D) a TUNEL assay was used to detect apoptosis; (E) Fluo‐3 AM assay was used to detect cellular Ca^2+^ levels. ^*^ *p <* .05 and ^**^ *p <* .01.

Two VIP siRNA fragments were constructed, and they both significantly reduced VIP levels. Because si‐VIP‐2 had the most significant knockdown level of VIP, we utilized si‐VIP‐2 in subsequent experiments (Figure 4A). According to the ELISA results, the upregulation of miR‐19a increased VIP levels, and the knockdown of VIP decreased VIP levels. Moreover, the enhancing effect of miR‐19a on VIP was reversed by VIP knockdown (Figure 4B). The CCK‐8 assay indicated that ICC viability was decreased by miR‐19a upregulation, and that VIP knockdown significantly increased ICC viability (Figure 4C). Similarly, TUNEL staining showed that the upregulation of miR‐19a increased ICC apoptosis and that the knockdown of VIP inhibited ICC apoptosis (Figure 4D). According to the Fluo‐3 AM assay, the Ca^2+^ concentration was decreased by miR‐19a upregulation in ICCs, whereas the knockdown of VIP significantly increased the Ca^2+^ concentration in ICCs (Figure 4E). The inhibitory influence of high miR‐19a levels on cell proliferation, anti‐apoptosis effects, and Ca^2+^ concentration in ICCs was reversed by VIP downregulation (Figure 4C–E). Thus, these findings indicated that miR‐19a affects ICCs through the upregulation of VIP.

### 3.5 VIP inhibits ICC activity via the NO–cGMP–PKG pathway

**FIGURE 5:**
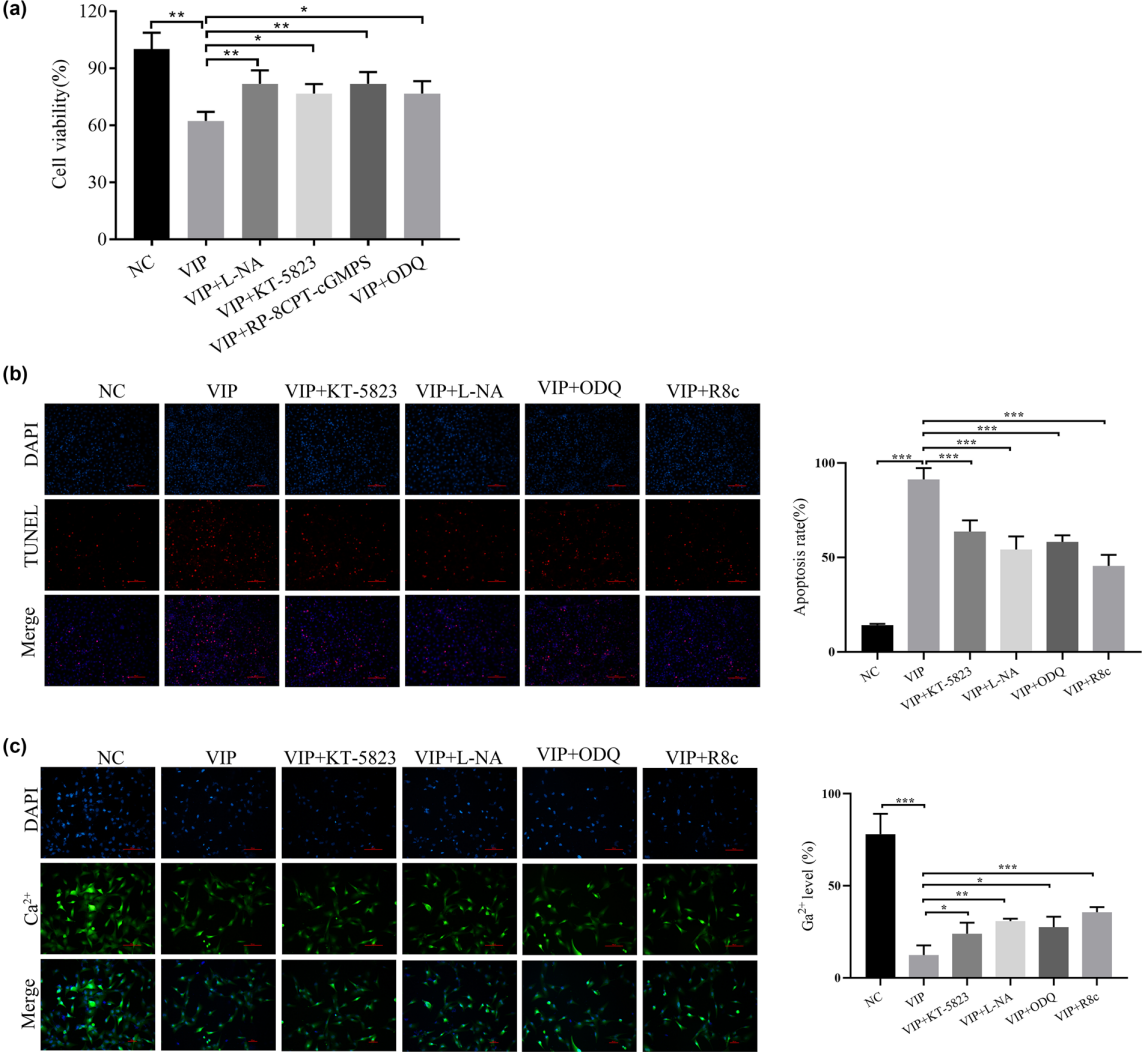
VIP affects interstitial cells of Cajal (ICCs) through the NO–cGMP–PKG pathway: (A) detection of cell viability by CCK‐8 assay; (B) a TUNEL assay was used to detect apoptosis; (C) a Fluo‐3 AM assay was used to detect cell Ca^2+^ concentration. ^*^ *p <* .05 and ^**^ *p <* .01.

To test whether the effect of VIP on ICCs is dependent on the NO–cGMP–PKG pathway, we used VIP together with a nonselective NOS inhibitor (L‐NA), PKG inhibitors (KT‐5823 and RP‐8CPT‐cGMPS), and a guanylate cyclase inhibitor (ODQ) to treat ICC cells. The results showed that L‐NA, KT 5823, RP‐8CPT‐cGMPS, and ODQ all partially restored the inhibitory effects of VIP on ICC cell proliferation, anti‐apoptosis effects, and Ca^2+^ concentration (Figure 5A–C). Thus, the effect of VIP on ICCs is partially dependent on the NO–cGMP–PKG pathway.

### 3.6 miR‐19a knockdown inhibits GID in TBI rats

**FIGURE 6:**
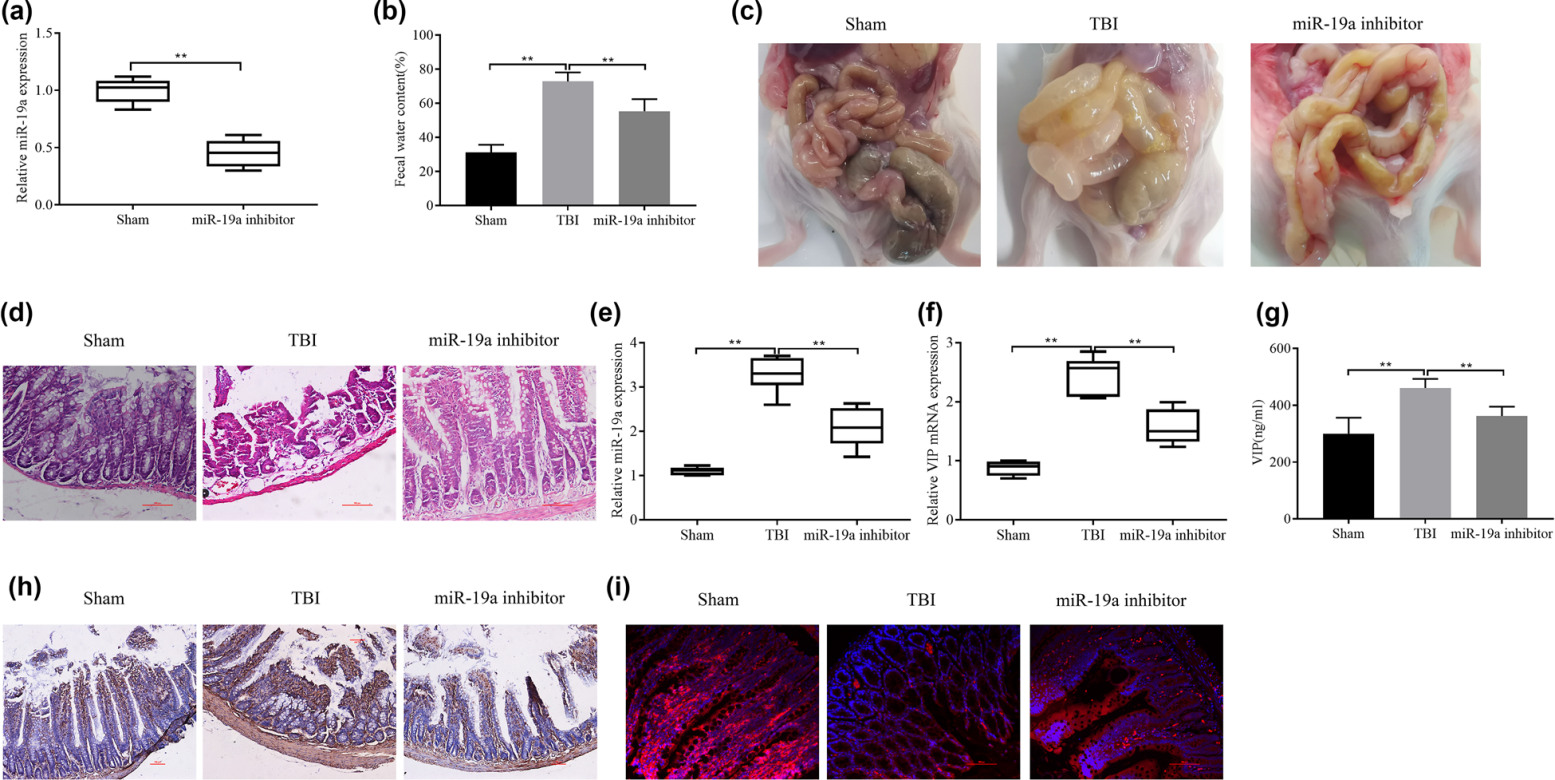
miR‐19a knockdown inhibits gastrointestinal dysfunction (GID) in traumatic brain injury (TBI) rats: detection of the transfection efficiency of the miR‐19a inhibitor by qRT‐PCR (A) and the effect of miR‐19a inhibition on the levels of miR‐19a (E) and VIP mRNA (F) after TBI; (B) macroscopic map of rat gastrointestinal tract morphology; (C) detection of rat fecal water content; (D) HE staining was utilized to observe intestinal histopathological features; (G) ELISA analysis of VIP levels in serum; (H) immunohistochemistry of VIP expression in ileal tissues; (I) immunofluorescence analysis of c‐kit expression in ileal tissues. ^*^ *p <* .05 and ^**^ *p <* .01.

The miR‐19a inhibitor was administered to rats by intraperitoneal injection, and the inhibitor efficiency was confirmed by qRT‐PCR analysis of serum miR‐19a expression levels 48 h after femoral artery blood collection. miR‐19a expression was significantly reduced in the miR‐19a inhibitor group (Figure 6A). After successful miR‐19a inhibition, the rats were subjected to CCI, and the fecal water content was measured after 72 h. The increase in fecal water content caused by TBI was inhibited by the miR‐19a inhibitor (Figure 6B). Opening the abdominal cavity to observe the gastrointestinal condition revealed that the miR‐19a inhibitor suppressed TBI‐induced gastrointestinal dilatation, yellowish fluid accumulation, and gastrointestinal thin wall edema (Figure 6C). Both abnormal intestinal wall morphogenesis and loss of intestinal structural integrity can result from damage and loss of intestinal villi. The miR‐19a inhibitor group showed recovered intestinal mucosa and reconstituted villi (Figure 6D). qRT‐PCR detection of serum miR‐19a and VIP mRNA expression in rats indicated that miR‐19a and VIP mRNA expression was reduced in the miR‐19a inhibitor group compared to the TBI group (Figure 6E,F). Similarly, the ELISA analysis of serum VIP levels demonstrated significantly lower VIP expression in the miR‐19a inhibitor group (Figure 6G). Immunohistochemistry was used to detect VIP expression in ileal tissues, which demonstrated that VIP expression was significantly increased in the TBI group and that the miR‐19a inhibitor had an inhibitory effect on VIP expression (Figure 6H). Immunofluorescence detection indicated that c‐kit expression was decreased in the TBI group, whereas c‐kit expression in the miR‐19a inhibitor group was enhanced (Figure 6I). Thus, these results indicated that miR‐19a knockdown inhibits GID in TBI rats.

## 4 DISCUSSION

ICCs are a group of cells that regulate gastrointestinal motility. Slow waves in small intestinal ICCs have been reported to depolarize smooth muscle cells and activate L‐type Ca^2+^ channels. Ca^2+^ entry through these channels triggers gastrointestinal muscle contractile activity, leading to stereotypic motor activity, such as peristalsis and segmentation (Youm et al., 2019; Zhang et al., 2018). Blockade of store‐operation calcium entry and inhibition of both external and internal Ca^2+^ eliminates ICC pacemaker potential (Kim et al., 2017; Park et al., 2018; Youm et al., 2019). Impairment and a reduced number of ICCs have been found in many GIDs, for example, gastroparesis (Grover et al., 2019), constipation (Zheng et al., 2021), and diarrhea (Jang et al., 2018). Because c‐kit is a transmembrane protein expressed in almost all ICCs, it has become an important marker for detecting the presence of CCI (Liu et al., 2021). In the present study, after 72 h of TBI, rats showed significant gastrointestinal elongation with large amounts of yellowish fluid and gastrointestinal thin wall edema as well as reduced c‐kit expression, indicating a simultaneous decrease in the number of ICCs.

Numerous studies have shown that many signaling pathways are regulated by miRNAs, which are widely involved in the pathophysiology of a variety of gastrointestinal disease states (Law et al., 2017). For example, miRNA‐29a regulates intestinal barrier function in irritable bowel syndrome with diarrhea through the regulation of ZO‐1 and CLDN1 (Zhu et al., 2020). miR‐10b‐5p targets KLF11, which in turn regulates KIT expression and improves GID in diabetes and post‐diabetes (Singh et al., 2021). Inhibition of miR‐19a upregulates neurohypophyseal gastrin levels to improve constipation (Wang et al., 2021). In addition, miR‐19a levels are significantly upregulated in rat serum 48 h after TBI (Bhomia et al., 2016). In the present study, miR‐19a expression was upregulated in rat ileal tissue beginning at 48 h after TBI with peak miR‐19a expression at 72 h after TBI. Moreover, the inhibition of miR‐19a improved diarrhea after TBI. Our results found an interesting phenomenon that the expression of VIP mRNA in serum detected by RT‐qPCR and VIP level in serum detected by ELISA showed inconsistent expression, and the possible reason was analyzed to be inconsistent transcription and translation of VIP gene. This speculation still needs to be confirmed by a large number of experiments. In vitro experiments demonstrated that miR‐19a overexpression decreased the cell viability, increased apoptosis, and reduced Ca^2+^ levels of ICCs.

Changes in the expression of brain and intestinal peptides, such as VIP, cholecystokinin, calcitonin gene‐related peptide, gastrin, and substance P, in the blood circulation and related tissues after TBI lead to the development of symptoms, such as bloating and diarrhea (Grundy et al., 2001; Kirkup et al., 2001). VIP is one of the most abundant neuropeptides innervating the gastrointestinal tract in several mammalian species, and it is expressed in several enteric neuron subtypes, most notably submucosal secretory motor neurons (Yakabi et al., 2018). VIP is now widely accepted as a major mediator of watery diarrhea syndrome (Chey et al., 2017), and VIP overexpression leads to secretory diarrhea (Sintusek et al., 2017). In the present study, miR‐19a upregulated VIP levels in ICCs, and VIP knockdown increased the cell viability, inhibited apoptosis, and increased Ca^2+^ levels of ICCs. At the same time, animal experiments found that the serum VIP level of TBI rats increased, and the increased VIP concentration may be the cause of intestinal dilation and large amounts of intestinal effusion.

NO is mainly produced during biosynthesis catalyzed by NOS, and it activates soluble guanylate cyclase, which increases cyclic guanosine monophosphate concentration, leading to increased phosphorylation and excitability of certain membrane proteins of cGMP‐dependent protein kinase (PKG) (Plano et al., 2021; Wu et al., 2020). It has been reported that the inhibition of the NO‐cGMP‐PKG pathway attenuates visceral hypersensitivity in rats in a functional dyspepsia model (Wu et al., 2020). The NO‐cGMP‐PKG pathway has also been shown to be activated by VIP (Grider & Murthy, 2008; Kim et al., 2006). The present study showed that VIP activated the NO‐cGMP‐PKG pathway and that a nonselective NOS inhibitor (L‐NA), PKG inhibitors (KT‐5823 and RP‐8CPT‐cGMPS), and guanylate cyclase inhibitor (ODQ) reversed the influence of VIP on the proliferation, apoptosis, and Ca^2+^ levels of ICCs.

In summary, the present study demonstrated that miR‐19a expression is elevated, and VIP expression is upregulated after TBI, thereby reducing the number of ICCs through the NO–cGMP–PKG pathway and suppressing Ca^2+^ levels, leading to gastrointestinal dysmotility diarrhea. In conclusion, miR‐19a is a key regulatory gene in TBI, and this discovery may provide certain theoretical basis for the treatment of TBI.

## AUTHOR CONTRIBUTIONS

**Ying An, Wenjun Ren, and Sheng Hu**: Conceptualization. **Ying An, Sheng Hu, and Wenjun Ren**: Methodology. **Sheng Hu and Yu Zhang**: Software. **Zhengji Song and Ruochang Li**: Validation. **Yan Li, Yongli Li, and Ping Wan**: Formal analysis. **Wenjun Ren, and Ping Wan**: Investigation. **Ping Wan**: Resources. **Sheng Hu, Zhengji Song, and Yan Li**: Data curation. **Ying An, Yu Zhang, and Ruochang Li**: Writing—original draft preparation. **Wenjun Ren, Yongli Li, and Ping Wan**: Writing—review and editing. **Sheng Hu and Ping Wan**: Visualization. **Ping Wan**: Supervision. **Ping Wan, Ying An, and Wenjun Ren**: Funding acquisition. All authors have read and agreed to the published version of the manuscript.

## CONFLICT OF INTEREST STATEMENT

The authors have no conflict of interests to declare.

### PEER REVIEW

The peer review history for this article is available at https://publons.com/publon/10.1002/brb3.3071.

## Data Availability

The data in the current study are available from the corresponding author on reasonable request.
